# Current Change Rate Influences Sensorimotor Cortical Excitability During Neuromuscular Electrical Stimulation

**DOI:** 10.3389/fnhum.2019.00152

**Published:** 2019-05-15

**Authors:** Sheng-Long Jiang, Zhongpeng Wang, Weibo Yi, Feng He, Hongzhi Qi, Dong Ming

**Affiliations:** ^1^Biomedical Engineering Department, School of Precision Instrument & Opto-Electronics Engineering, Tianjin University, Tianjin, China; ^2^Beijing Machine and Equipment Institute, Beijing, China; ^3^Academy of Medical Engineering and Translational Medicine, Tianjin University, Tianjin, China

**Keywords:** neuromuscular electrical stimulation (NMES), current change rate, cortical excitability, time-frequency domain features, source analysis

## Abstract

Neuromuscular electrical stimulation (NMES) is frequently used in rehabilitation therapy to improve motor recovery. To optimize the stimulatory effect of NMES, the parameters of NMES, including stimulation mode, location, current intensity, and duration, among others have been investigated; however, these studies mainly focused on the effects of changing parameters in the current plateau stage of the NMES cycle, while the impacts on other stages, such as the current rising stage, have yet to be investigated. In this article, we studied the electroencephalograph (EEG) effects during NMES, with different rates of current change in the rising stage, and stable current intensity in the plateau stage. EEG signals (64-channel) were collected from 28 healthy subjects, who were administered with high, medium, or low current change rate (CCR) NMES through a right-hand wrist extensor. Time-frequency analysis and brain source analysis, using the LORETA method, were used to investigate neural activity in sensorimotor cortical areas. The strengths of cortical activity induced by different CCR conditions were compared. NMES with a high CCR activated the sensorimotor cortex, despite the NMES current intensity in the plateau stage lower than the motor threshold. Reduction of the Alpha 2 band (10–13 Hz) event related spectral power (ERSP) during NMES stimulation was significantly enhanced by increasing CCR (*p* < 0.05). LORETA-based source analysis demonstrated that, in addition to typical sensory areas, such as primary somatosensory cortex (S1), sensorimotor areas including primary motor cortex (M1), premotor cortex (PMC), and somatosensory association cortex (SAC) were all activated by within threshold NMES. Furthermore, compared with the low CCR condition, cortical activity was significantly enhanced in the S1, M1, and PMC areas under high CCR conditions. This study shows CCR in the NMES rising stage can affect EEG responses in the sensorimotor cortex and suggests that CCR is an important parameter applicable to the optimization of NMES treatment.

## Introduction

Neuromuscular electrical stimulation (NMES) has been widely used in the clinical treatment of motor dysfunction for many years. Based on the review of Chipchase et al. (2011) NMES studies includes both functional electrical stimulation (FES) and therapeutic electrical stimulation (TES). Induction of muscle contractions by NMES to supplement or substitute for lost motor functions is referred to as FES (Doucet et al., 2012). A typical application of FES is to help patients with spinal cord injury (SCI) to regain the ability to grasp, hold, and release objects (Rupp et al., 2012; Thorsen et al., 2014). For central nervous system diseases, task-related activation method on brain functional areas is as important as motor assistance for rehabilitation. Some of the studies focus on brain activation induced by NMES which played an important role of rehabilitation in the central nervous system injury. Wegrzyk et al. (2017) contrasted specific brain activation patterns associated with wide-pulse high-frequency (100 Hz–1 ms) and conventional (25 Hz–0.05 ms) NMES. It is worth mentioning that the human brain is highly plastic during development as new connections are formed through task-related processes. Increased excitability in surviving neurons might leads to enhanced motor recovery after stroke (Murphy and Corbett, 2009).

Neural image studies reported NMES elicited cortical activation across a wide network of cortical and subcortical structures, similar to that activated during repeated isometric voluntary contractions. A functional magnetic resonance imaging (fMRI) study showed significantly different cortical activity in the cerebellar and secondary somatosensory areas between NMES movement and voluntary movement (Iftime-Nielsen et al., 2012). Moreover, using near-infrared spectroscopy (NIRS), a redistribution of cortical brain perfusion was identified in the ipsilesional SMC during the electrical stimulation (Hara et al., 2013).

Although questions remain regarding the neural mechanisms underlying the influence of NMES on cortical reorganization, many studies have been conducted to optimize NMES treatment by varying stimulatory factors, such as the current intensity, frequency, pulse duration and location of NMES (Quandt and Hummel, 2014; Obiglio et al., 2016). The intensity of the stimulation current has also been proven to correlate with cortical excitation, with higher motor evoked potential (MEP) detected during higher intensity stimulation (Sasaki et al., 2017). But, Muthalib et al. (2015) reported greater bilateral sensorimotor network activation profile with high NMES current intensities could be in part attributable to increased attentional/pain processing. Moreover, it has been reported that a low-intensity sensory threshold NMES can also induce significant activation over sensorimotor areas and enhance brain connectivity patterns without artifacts (Corbet et al., 2018), and, low-intensity NMES showed treatment effects similar to high-intensity NMES in stroke patients (Hsu et al., 2010).

To date, the majority of studies on NMES parameters have focused primarily in the stimulation plateau stage and, to our knowledge, there are no reports of investigations of other stages, such as the stimulation current rising stage. In this article, we discuss cortical excitability change to the current changing rate (CCR) in the rising stage of NMES. Our hypothesis was the different CCRs in NMES rising stage would induced variations in sensorimotor cortical activation during NMES with the same current intensity (i.e., pulse amplitude) in the plateau stage and an increasing CCR induced stronger cortical activation even with the less total current charge.

## Materials and Methods

### Experiment

**Figure 1:**
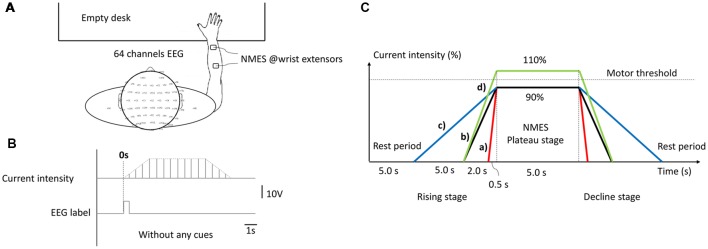
The experimental configuration. **(A)** Experimental set-up. **(B)** Current intensity curve during one neuromuscular electrical stimulation (NMES) trial. **(C)** Temporal structure of stimulation under different conditions, as follows: (a) H-WT; (b) M-WT; (c) L-WT and (d) M-ET.

Healthy right-handed graduate students (*n* = 28), aged 22.89 ± 1.663 years were included in this study. All subjects were seated in a comfortable armchair in front of an empty table. During the experiment, subjects were required to remain in a resting state and avoid any unnecessary motion. NMES was applied over the central muscle belly of right-hand wrist extensors in the back of forearm with a pair of 4 * 4 cm square self-adhesive surface electrode placed at 4 cm intervals (presented in Figure 1A), which was implemented by an FDA approved electrical stimulator (Intelect^®^ Legend XT NMES system, Chattanooga, USA). The basic NMES parameters were a constant-current mode of 30 Hz and a 200 μs pulse duration symmetrical biphasic square-pulsed current (presented in Figure 1B), which widely used in rehabilitation of central nervous system diseases such as post-stroke hemiplegia and paraplegia after SCI (Gregory et al., 2007).

The experimental design is presented in Figure 1C. Three types of CCR were used, designated high, medium, and low. Under these three CCR conditions, the intensity of the NMES stimulation current increased from 0 to plateau intensity after 0.5 s (high), 2 s (medium), and 5 s (low), respectively. Because different individuals respond differently to the same current intensity of NMES, in order to eliminate individual difference and avoid painful sensations, we use a relative value of “motor threshold” instead of an absolute value of “current intensity”. Furthermore, “motor threshold” is a conventional benchmark for NMES current intensity in many previous studies (Boisgontier et al., 2009; Lim et al., 2014). We set two types of plateau period intensity: within motor threshold (90% current, WT) and exceeding motor threshold (110% current, ET). We increased the NMES intensity by a step of 0.5 mA and stopped at a subject-specific motor threshold by a visible muscle contraction. Before we placed electrodes, subjects’ right forearm skin was cleaned. According to the records, the maximum NMES intensities of each subject were below 15 mA. Four experimental conditions were used: 0.5 s rising time and 90% threshold intensity (H-WT); 2 s rising time and 90% threshold intensity (M-WT); 5 s rising time and 90% threshold intensity (L-WT); and 2 s rising time and 110% threshold intensity (M-ET). The execution sequence of four sessions was random, and rest duration of each session was 5 mins. The primary focus of this study was the effect of variation in CCR under the WT condition, the ET condition was included as a control. Because we worried that if the subjects tried to distinguish between stimulation conditions would elicit extra neural activity such as attention and cognitive behavior, we did not require subjects to distinguish them. At the end of the experiment, we recorded the subjects’ feelings. Based on the survey, most of the subjects indicated that they were not sensitive to the change of CCR.

The experiment comprised four different sessions, each of which consisted of 25 randomized controlled trials under H-WT, M-WT, L-WT, and M-ET conditions. Each trial included four stages: resting, current rising, plateau, and current falling. The duration of the rest and plateau stages were 5 s each. The duration of rising and decline stages were the same as one another; however, they differed according to the experimental conditions. For example, in an M-WT trial, the rising and falling stage were both 2 s. The experimental protocol was approved by the Ethical Committee of Tianjin University.

### EEG Recording

EEG signals were recorded using a Neuroscan SynAmps RT EEG amplifier with a 64-channel quick-cap and the International 10/20 system. The reference electrode and ground electrode were placed on the right and left ear lobes, respectively. The impedance of the electrodes was maintained at <5 KΩ. EEG signals were acquired at a sampling rate of 1,000 Hz and band-pass filtered between 0.05 and 100 Hz. A 50 Hz notch filter was used during data acquisition. Before data analysis, raw EEG signals were down-sampled to 256 Hz.

### Data Analysis

In this study, we focused on the effects of CCR on cortical excitability, especially sensorimotor regions, so we used standard methods of channel time-frequency maps and source analysis (Gwin and Ferris, 2012; Iftime-Nielsen et al., 2012).

The mean spectral power changes related to NMES in a time-frequency domain were visualized using the event-related spectral perturbation (ERSP), which provides detailed information on spectral power neural oscillation during different tasks (Makeig, 1993; Delorme and Makeig, 2004). The ERSP of *n* trials was calculated according to equation (1), as follows:

(1) $$ERSP(f,t)=\frac{1}{n}\sum_{k=1}^{n}\left(F_{k}{\left(f,t\right)}^{2}\right)$$

where *n* is the number of trials, and *F* is the spectral estimation of the kth trial at frequency f and time t. EEGLAB with MATLAB was used to compute the ERSP (dB) through short-time Fourier transform (STFT) with a Hanning-tapered window (length, 256 points). To produce the baseline-normalized ERSP, the mean power changes in a baseline period (2 s before applying NMES) were subtracted from each spectral estimation. For analysis, ERSP maps from the C3 electrode were displayed from 2 s before to 2 s after the NMES between 8 and 32 Hz. Hereafter, ERSP refers to baseline-normalized ERSP.

The ERSP values of different CCR conditions were compared in five corresponding event-related desynchronization (ERD) frequency bands: alpha1 (8–10 Hz), alpha2 (10–13 Hz), beta1 (13–18 Hz), beta2 (18–24 Hz), beta3 (24–30 Hz), and a single somatosensory steady-state evoked potential (SSSEP) corresponding frequency band, 29–31 Hz. Prior to comparisons, ERSP values were averaged over the entire stimulation plateau period and frequency band.

In addition, the precise low-resolution brain electromagnetic tomography (LORETA) approach was used to compute cortical current density distributions from scalp EEG readings (Pascual-Marqui et al., 2011). The LORETA method is widely used to evaluate active and resting states of cortex regions. In this study, 6,239 cortical voxels of spectral density were computed using the MNI152 2009c T2 template from a 60-channel scalp EEG.

Sensorimotor integration can be thought of in terms of input-output systems. To analyze the functional activation in cortical sensorimotor areas during NMES, we chose contralateral cortical sensorimotor areas, following Brodmann areas (BA) as regions of interest (ROIs). We defined five ROIs including the premotor cortex (PMC, BA 6), the primary motor cortex (M1, BA 4), the primary somatosensory cortex (S1, BA 3–1–2), the secondary somatosensory cortex (S2, BA 43), and the somatosensory association cortex (SAC, BA 5 and 7) and performed eLORETA source analysis. Two aspects of the activation in each ROI were evaluated: (1) the number of active voxels in each ROI; and (2) the average current density value of all active voxels in each ROI. The former measures the active range in the ROI and the latter determines the strength of activation of these voxels.

For statistical analysis, we used one-way analysis of variance (ANOVA) to compare the effects of CCR among the three WT conditions by SPSS 20 (SPSS Inc., Chicago, IL, USA). If statistical results conformed to the assumptions of normal distribution (one-sample Kolmogorov-Smirnov test), homogeneity (Student-Newman-Keuls method) and ANOVA results conformed to significant difference between group, we performed comparison of ERSP and the cortical activation in ROIs between different conditions by pairwise *t*-tests with Bonferroni correction (pairs = 2, alpha value = 0.05, tail = 0, by MATLAB 2014b).

## Results

### Event Related Spectral Power

**Figure 2:**
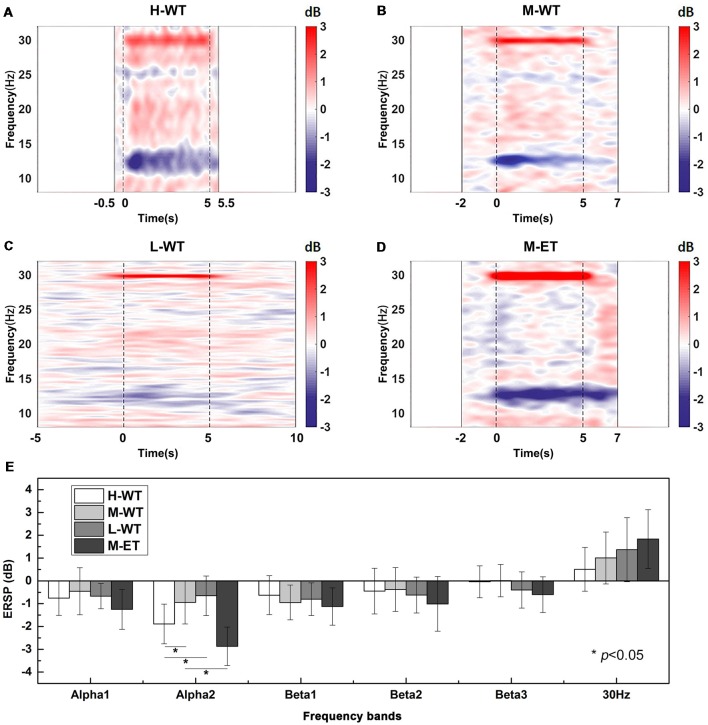
Averaged C3 channel time-frequency maps during NMES under different conditions and statistical analyses. **(A)** H-WT, **(B)** M-WT, **(C)** L-WT, and **(D)** M-ET conditions. Red color bar means band electroencephalograph (EEG) synchronization patterns (ERS) and blue means EEG desynchronization patterns (ERD). **(E)** Comparison among four conditions of event related spectral power (ERSP) at different frequency bands. *p < 0.05.

The results of EEG time-frequency analysis at the C3 electrode are presented in Figure 2. When the NMES current exceeded the motor threshold the EEG oscillations in the alpha band were clearly depressed (Figure 2D), consistent with numerous previous studies (Blickenstorfer et al., 2009; Jang et al., 2014). However, even with a stimulation current intensity lower than the motor threshold, the alpha band EEG oscillation was also depressed during NMES (Figures 2A–C). These results indicate that the ERD alpha band can be induced by NMES within the motor threshold.

In particular, the CCR significantly influenced the ERD strength, with high CCR inducing a marked inhibition of alpha band oscillation, while low CCR only led to a slight depression. One-way ANOVA analysis showed that the ERSP varied significantly among the three CCR conditions (*F* = 8.261, *p* = 0.020). Pairwise *t*-test with Bonferroni correction also demonstrated that the ERSP depression under H-WT was significantly greater than that under M-WT (*p* = 0.021, Cohen’s *d* = 1.040) and L-WT (*p* = 0.017, Cohen’s *d* = 1.424; Figure 2E).

As expected, although WT NMES induced alpha band ERD, the induction was weaker than that induced by ET NMES. Under the same CCR conditions, the ERSP reduction under M-ET was significantly higher than that under M-WT (*p* = 0.014, Cohen’s *d* = 2.152; Figure 2E). These results demonstrate that not only the intensity of the NMES current in the plateau stage but also the CCR in the rising stage, are important influences on alpha band EEG oscillation. As there are no previous publications that report the influence of F on alpha band ERD, this result suggests a new factor that can be used to optimize NMES treatment.

Another interesting phenomenon observed was the spectral power enhancement around 30 Hz (Figures 2A–D). As we can see, the time-frequency maps in Figures 2A–C showed that the frequency bandwidth of the SSSEP around 30 Hz was different and the 30 Hz ERSP in Figure 2E is different, which suggested that this 30 Hz neural oscillation phenomenon is a neural response. And, the previous study reported similar neural oscillation phenomena of SSSEP (Müller-Putz et al., 2006). Clearly, this enhancement corresponds to the SSSEP induced by periodic electrical stimulation at 30 Hz; however, unlike the ERD alpha band phenomenon, the amplitude of the SSSEP differed significantly according to current intensity, but not CCR. Bonferroni corrected pairwise *t*-test indicated that the SSSEP amplitude in M-ET was no difference with M-WT (*p* = 0.052); and, there was no significant difference among the WT conditions (*F* = 7.218, *p* = 0.076). This suggests that, compared with the simple sensory stimulus responses in SSSEP, the ERD alpha band involves a more complex neural response mechanism.

### Cortical Activity

**Figure 3:**
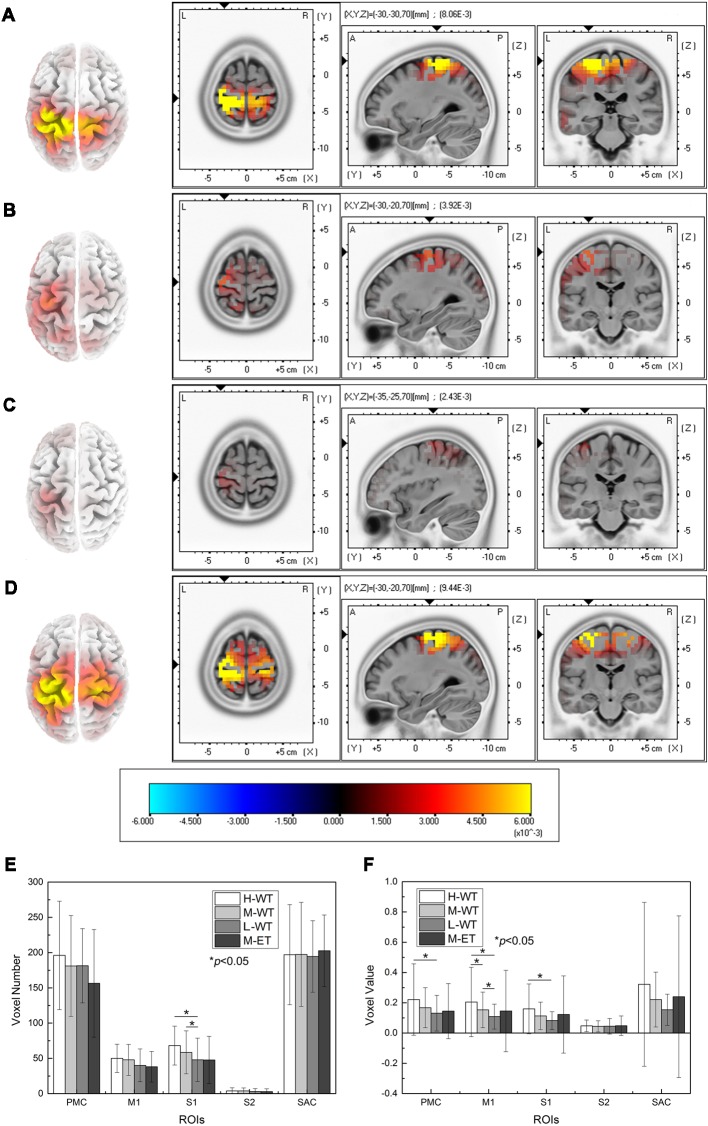
Cortical activity determined using the LORETA method and statistical analysis of the results. **(A)** H-WT, **(B)** M-WT, **(C)** L-WT, and **(D)** M-ET conditions. **(E)** Comparison of the active ranges under different conditions in five cortical regions. **(F)** Comparison of the intensity of activity in five regions under different conditions.

To further evaluate the cortical neural activity, we used the LORETA method to perform EEG source analysis. The cortical areas activated under different experimental conditions are illustrated in Figures 3A–D. First, similar to the results presented in Figure 2, cortical activation occurred during WT NMES. Cortical activation in the H-WT condition was much stronger than that in the L-WT conditions, both in terms of activation range and activation intensity. In particular, both sides of the sensorimotor area were activated under H-WT conditions, in a similar way to that observed during ET stimulation; however, only one side of the sensorimotor area was activated under M-WT and L-WT conditions. These results suggest that comparable cortical activation can be induced in the absence of actual limb activity.

Further comparisons among the effects of different NMES conditions on five areas (S1, M1, S2, PMC, and SAC) are presented in Figures 3E,F. In S1, a significant difference was identified among WT conditions in terms of both activated volume and activation intensity (*F* = 6.992, *p* = 0.038; *F* = 8.439, *p* = 0.027). Compared with the L-WT condition, the average activation voxel number in the S1 area increased significantly by 42% under H-WT (*p* = 0.012, Cohen’s *d* = 0.689) and by 22% under M-WT (*p* = 0.024, Cohen’s *d* = 0.343). These phenomena correspond well with the reported perception of the subjects since variations in CCR lead to distinctly different sensations.

Notably, activation intensity change was also observed between the H-WT and L-WT conditions in the M1 and PMC areas. Compared with the L-WT condition, the average voxel value in the M1 area increased by 89% (*p* = 0.011, Cohen’s *d* = 0.559) and 41% (*p* = 0.017, Cohen’s *d* = 0.436) under H-WT and M-WT conditions, respectively. And, compared with the L-WT condition, the average voxel value of the PMC area increased significantly 69% (*p* = 0.020, Cohen’s *d* = 0.485) the average voxel value of the S1 area increased significantly 94% (*p* = 0.010, Cohen’s *d* = 0.627) under H-WT conditions. These results confirm that the motor-related cortex response a stronger activation to high CCR. As NMES is widely used for post-stroke rehabilitation, these findings provide an additional option for optimization of NMES treatment.

No significant difference in cortical activity was found between the H-WT and the M-ET conditions, despite an actual movement occurring under the M-ET condition. This indicates that CCR is a relatively strong influence on cortical activity during NMES and that it is unnecessary to induce actual passive limb movement to induce comparable cortical activation.

**Figure 4:**
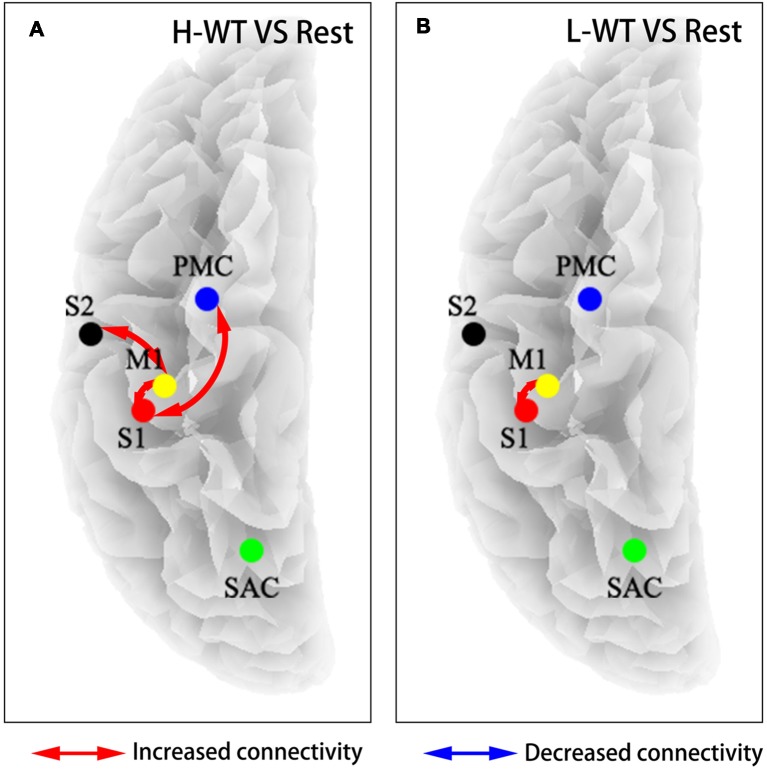
Flow connectivity between sensorimotor areas under different conditions. **(A)** NMES with H-WT condition promoted connections between multiple brain functional areas when compared with non-stimulation; **(B)** NMES with L-WT condition promoted connection between M1 and S1 areas when compared with non-stimulation.

To further investigate the cooperation between neural activities among sensorimotor areas, we use partial directed coherence (PDC) to evaluate information transfer among the M1, S1, S2, and PMC regions. Figure 4 shows the connectivity among these areas under H-WT and L-WT conditions. Under H-WT conditions, NMES induced a significant correlation between M1 and S1, M1 and S2, and PMC and S1 (presented in Figure 4A), while under L-WT conditions only M1 and S1 exhibited significant correlation (presented in Figure 4B). These data may indicate that more complex sensory-motor cooperation occurs when a high CCR is applied, despite the same current intensity at the platform stage.

## Discussion

Previous studies aiming to optimize NMES treatments have focused primarily on location, intensity, duration, or pulse patterns (Lagerquist and Collins, 2010; Muthalib et al., 2015; Cometti et al., 2016). To our knowledge, no previous reports have discussed the influence of the rising rate of stimulus current during NMES. Based on cortical excitability data, this study showed the CCR induced different sensorimotor rhythm neural activity in the S1, M1, and PMC regions, with particularly marked variation between the high CCR and low CCR conditions. This kind of task-related activities in the sensorimotor areas plays a positive role in the rehabilitation therapy of patients with central nervous system injury (Wegrzyk et al., 2017). A possible reason for these observations is that the high current variation during the rising stage under H-WT conditions induces a strong sensory stimulus which excites the associated sensory channel.

Numerous clinical studies have demonstrated that NMES treatment during rehabilitation can induce neural plasticity changes, leading to improvements in voluntary motor control. Neural imaging studies show that both the motor cortex and the wider sensorimotor cortex are activated during the passive movement induced by NMES (An et al., 2013). Additionally, even where there is no passive motor induction, neural plasticity changes can be induced by sensory stimulation or sensory feedback, such as visual perception. However, the question of how NMES sensory stimulation induces cortical reorganization remains open; multiple mechanisms have been proposed, including mirror neurons, antidromic firing of motor nerve fibers (Rushton, 2003), and motor learning. Currently, the consensus view is that sensory and motor stimulation can promote athletic rehabilitation. This study demonstrates that it is possible to induce stronger cortical activity in sensorimotor areas by choosing an appropriate CCR, suggesting that CCR is a factor that could be optimized to improve NMES treatment.

During neural rehabilitation, the induction of plastic changes to the central nervous system is one of the most important considerations. A reliable evaluation of neural plastic changes should be based on the long-term effects of clinical rehabilitation, which undoubtedly requires high levels of clinical resources and significant time. However, numerous studies have shown that there is a high positive correlation between the effect of clinical rehabilitation and cortical excitability during treatment (Murphy and Corbett, 2009; Johansson, 2011). In other words, cortical excitability can be used as an indicator to facilitate optimization and development of rehabilitation strategies. Many physiological measurements can be used to evaluate cortical excitability, including fMRI, fNIRS, and TMS induced MEP (Bastani and Jaberzadeh, 2012; Beaumont et al., 2014; Nardone et al., 2015). However, EEG based cortical excitability measurement provides the specific advantage of high temporal resolution, which allows real-time detection during NMES treatment. Moreover, the use of an electrical current in the MRI environment introduces a potential risk of injury, and it is difficult to evaluate the cortical activity in such a wide area of the sensorimotor cortex during TMS induced MEP.

The NMES evoked central nervous system activation has been demonstrated by many previous studies, which have a clear rehabilitation effect for post-stroke hemiplegia (Smith et al., 2003; Wegrzyk et al., 2017). However, continuous high current NMES may cause negative effects such as muscle fatigue, even skin burn (Gorgey et al., 2009). One potential solution is to use multiple channel electrical stimulation to recruit motor units following a predefined order (Reynolds et al., 2015). Another option is to reduce the current of electrical stimulation to reduce the potential risk of secondary damage caused by incorrect high-intensity stimulation, because sensory impairment in motor dysfunction patients may unable to accurately determine the maximum stimulation intensity they can accept (Dudley-Javoroski and Shields, 2008; Sayenko et al., 2014). Clearly, it is necessary to balance the muscle fatigue with therapeutic effects. And, a low-intensity NMES showed treatment effects similar to high-intensity NMES in stroke patients (Hsu et al., 2010). Although low-intensity stimulation may reduce muscle activation, task-related activation of brain functional areas is more important. For brain damage patients, task-related cortical excitability can induce neural plasticity and functional recovery (Grosse-Wentrup et al., 2011; Ethier et al., 2015). If we use cortical excitability enhancement as the optimization parameter of NMES therapy, it may be able to improve the therapeutic effect for brain damage patients. The results of cortical excitability in the sensorimotor areas during WT NMES in this and other previous studies suggest that NMES parameters could be regulated to maintain cortical excitability while significantly reducing the current of electrical stimulation (Wegrzyk et al., 2017). Further investigations using diverse neural imaging techniques are required, along with clinical validation studies. However, the change of CCR does not conflict with NMES intensity adjustment.

With constant maximum current, pulse duration, and frequency, the increase of total current charge meant that the sensorimotor nervous system received more stimulation, which logically seems to induce a stronger neural response. However, in our study, the total current charge under L-WT condition was the largest, but it actually induced the lowest sensorimotor cortical excitability in the plateau stage, while H-WT condition induced the highest cortical excitability with the lowest total current. Hindle reported increasing pulse duration may not be an effective strategy for increasing cortico-spinal excitability for rehabilitation (Lagerquist et al., 2012). In our study, we got some similar results, L-WT condition has a larger total current charge than H-WT, but it did not enhance cortical excitability. According to these results, increasing CCR induced stronger cortical activation even with the decrease of total current charge, which further proved the core effect of CCR in promoting cortical excitability.

## Conclusion

In summary, this study demonstrates that the CCR in the rising stage during NMES has a significant influence on the cortical activity of sensorimotor areas by EEG analysis. High CCR NMES can evoke considerable excitability in these cortical areas, despite the use of a current intensity insufficient to cause limb movement execution. The results of this study provide new insights to facilitate optimization of NMES stimulation during rehabilitation treatment.

## Ethics Statement

This study was carried out in accordance with the recommendations of guidelines stated in the Declaration of Helsinki. The protocol was approved by the ethical committee of Tianjin University. All subjects gave written informed consent in accordance with the Declaration of Helsinki.

## Author Contributions

HQ conceived and designed the study. S-LJ performed the experiments and analyzed the data. S-LJ, ZW and HQ drafted and revised the manuscript. WY participated in experiments. FH participated in data analysis. DM participated in final version approval.

## Conflict of Interest Statement

The authors declare that the research was conducted in the absence of any commercial or financial relationships that could be construed as a potential conflict of interest.

## References

[B1] An J. Jin S. H. Lee S. H. Jang G. Abibullaev B. Lee H. (2013). “Cortical activation pattern for grasping during observation, imagery, execution, FES, and observation-FES integrated BCI: an fNIRS pilot study,” in Proceedings of the 2013 35th Annual International Conference of the IEEE Engineering in Medicine and Biology Society (Osaka: IEEE), 6345–6348. 10.1109/EMBC.2013.6611005 24111192

[B2] Bastani A. Jaberzadeh S. (2012). A higher number of TMS-elicited MEP from a combined hotspot improves intra- and inter-session reliability of the upper limb muscles in healthy individuals. PLoS One 7:e47582. 10.1371/journal.pone.0047582 23077645 PMC3471890

[B3] Beaumont E. Guevara E. Dubeau S. Lesage F. Nagai M. Popovic M. (2014). Functional electrical stimulation post-spinal cord injury improves locomotion and increases afferent input into the central nervous system in rats. J. Spinal Cord Med. 37, 93–100. 10.1179/2045772313y.0000000117 24090649 PMC4066556

[B4] Blickenstorfer A. Kleiser R. Keller T. Keisker B. Meyer M. Riener R.. (2009). Cortical and subcortical correlates of functional electrical stimulation of wrist extensor and flexor muscles revealed by fMRI. Hum. Brain Mapp. 30, 963–975. 10.1002/hbm.20559 18344193 PMC6870950

[B5] Boisgontier M. Vuillerme N. Thomas D. Pinsault N. Emprin M. Caillat-Miousse J. L. (2009). Effects of neuromuscular electrical stimulation on the range of motion recovery in hand proximal interphalangeal sprain. Sci. Sports 24, 192–195. 10.1016/j.scispo.2008.09.001

[B7000] Chipchase L. S. Schabrun S. M. Hodges P. W. (2011). Peripheral electrical stimulation to induce cortical plasticity: A systematic review of stimulus parameters. Clin. Neurophysiol. 122, 456–463. 10.1016/j.clinph.2010.07.025 20739217

[B6] Cometti C. Babault N. Deley G. (2016). Effects of constant and doublet frequency electrical stimulation patterns on force production of knee extensor muscles. PLoS One 11:e0155429. 10.1371/journal.pone.0155429 27167066 PMC4864221

[B7] Corbet T. Iturrate I. Pereira M. Perdikis S. Millán J. D. R. (2018). Sensory threshold neuromuscular electrical stimulation fosters motor imagery performance. Neuroimage 176, 268–276. 10.1016/j.neuroimage.2018.04.005 29689307

[B8] Delorme A. Makeig S. (2004). EEGLAB: an open source toolbox for analysis of single-trial EEG dynamics including independent component analysis. J. Neurosci. Methods 134, 9–21. 10.1016/j.jneumeth.2003.10.009 15102499

[B9] Doucet B. M. Lam A. Griffin L. (2012). Neuromuscular electrical stimulation for skeletal muscle function. Yale J. Biol. Med. 85, 201–215. 22737049 PMC3375668

[B10] Dudley-Javoroski S. Shields R. K. (2008). Muscle and bone plasticity after spinal cord injury: review of adaptations to disuse and to electrical muscle stimulation. J. Rehabil. Res. Dev. 45, 283–296. 10.1682/jrrd.2007.02.0031 18566946 PMC2744487

[B11] Ethier C. Gallego J. A. Miller L. E. (2015). Brain-controlled neuromuscular stimulation to drive neural plasticity and functional recovery. Curr. Opin. Neurobiol. 33, 95–102. 10.1016/j.conb.2015.03.007 25827275 PMC4523462

[B12] Gorgey A. S. Black C. D. Elder C. P. Dudley G. A. (2009). Effects of electrical stimulation parameters on fatigue in skeletal muscle. J. Orthop. Sports Phys. Ther. 39, 684–692. 10.2519/jospt.2009.3045 19721215

[B13] Gregory C. M. Dixon W. Bickel C. S. (2007). Impact of varying pulse frequency and duration on muscle torque production and fatigue. Muscle Nerve 35, 504–509. 10.1002/mus.20710 17230536

[B14] Grosse-Wentrup M. Mattia D. Oweiss K. (2011). Using brain-computer interfaces to induce neural plasticity and restore function. J. Neural Eng. 8:025004. 10.1088/1741-2560/8/2/025004 21436534 PMC4515347

[B15] Gwin J. T. Ferris D. P. (2012). An EEG-based study of discrete isometric and isotonic human lower limb muscle contractions. J. Neuroeng. Rehabil. 9:35. 10.1186/1743-0003-9-35 22682644 PMC3476535

[B16] Hara Y. Obayashi S. Tsujiuchi K. Muraoka Y. (2013). The effects of electromyography-controlled functional electrical stimulation on upper extremity function and cortical perfusion in stroke patients. Clin. Neurophysiol. 124, 2008–2015. 10.1016/j.clinph.2013.03.030 23706813

[B17] Hsu S. S. Hu M. H. Wang Y. H. Yip P. K. Chiu J. W. Hsieh C. L. (2010). Dose-response relation between neuromuscular electrical stimulation and upper-extremity function in patients with stroke. Stroke 41, 821–824. 10.1161/strokeaha.109.574160 20203321

[B18] Iftime-Nielsen S. D. Christensen M. S. Vingborg R. J. Sinkjaer T. Roepstorff A. Grey M. J. (2012). Interaction of electrical stimulation and voluntary hand movement in SII and the cerebellum during simulated therapeutic functional electrical stimulation in healthy adults. Hum. Brain Mapp. 33, 40–49. 10.1002/hbm.21191 21591025 PMC6870182

[B19] Jang S. H. Jang W. H. Chang P. H. Lee S. H. Jin S. H. Kim Y. G.. (2014). Cortical activation change induced by neuromuscular electrical stimulation during hand movements: a functional NIRS study. J. Neuroeng. Rehabil. 11:29. 10.1186/1743-0003-11-29 24597550 PMC3973889

[B20] Johansson B. B. (2011). Current trends in stroke rehabilitation. A review with focus on brain plasticity. Acta Neurol. Scand. 123, 147–159. 10.1111/j.1600-0404.2010.01417.x 20726844

[B21] Lagerquist O. Collins D. F. (2010). Influence of stimulus pulse width on M-waves, H-reflexes, and torque during tetanic low-intensity neuromuscular stimulation. Muscle Nerve 42, 886–893. 10.1002/mus.21762 20886511

[B22] Lagerquist O. Mang C. S. Collins D. F. (2012). Changes in spinal but not cortical excitability following combined electrical stimulation of the tibial nerve and voluntary plantar-flexion. Exp. Brain Res. 222, 41–53. 10.1007/s00221-012-3194-5 22899312

[B23] Lim K.-B. Lee H.-J. Yoo J. Kwon Y.-G. (2014). Effect of low-frequency rTMS and NMES on subacute unilateral hemispheric stroke with dysphagia. Ann. Rehabil. Med. 38, 592–602. 10.5535/arm.2014.38.5.592 25379488 PMC4221387

[B24] Makeig S. (1993). Auditory event-related dynamics of the EEG spectrum and effects of exposure to tones. Electroencephalogr. Clin. Neurophysiol. 86, 283–293. 10.1016/0013-4694(93)90110-h 7682932

[B25] Müller-Putz G. R. Scherer R. Neuper C. Pfurtscheller G. (2006). Steady-state somatosensory evoked potentials: suitable brain signals for brain-computer interfaces? IEEE Trans. Neural Syst. Rehabil. Eng. 14, 30–37. 10.1109/tnsre.2005.863842 16562629

[B26] Murphy T. H. Corbett D. (2009). Plasticity during stroke recovery: from synapse to behaviour. Nat. Rev. Neurosci. 10, 861–872. 10.1038/nrn2735 19888284

[B27] Muthalib M. Re R. Zucchelli L. Perrey S. Contini D. Caffini M.. (2015). Effects of increasing neuromuscular electrical stimulation current intensity on cortical sensorimotor network activation: a time domain fNIRS study. PLoS One 10:e0131951. 10.1371/journal.pone.0131951 26158464 PMC4497661

[B29] Nardone R. Höeller Y. Thomschewski A. Bathke A. C. Ellis A. R. Golaszewski S. M.. (2015). Assessment of corticospinal excitability after traumatic spinal cord injury using MEP recruitment curves: a preliminary TMS study. Spinal Cord 53, 534–538. 10.1038/sc.2015.12 25665538

[B30] Obiglio M. Mendelevich A. Jeffrey S. Drault E. Garcete A. Kramer M.. (2016). Peripheral nerve stimulation effectiveness in the upper limb function recovery of patients with a stroke sequel: systematic review and meta-analysis. Rev. Neurol. 62, 530–538. 10.33588/rn.6212.2015401 27270674

[B31] Pascual-Marqui R. D. Lehmann D. Koukkou M. Kochi K. Anderer P. Saletu B.. (2011). Assessing interactions in the brain with exact low-resolution electromagnetic tomography. Philos. Trans. A Math. Phys. Eng. Sci. 369, 3768–3784. 10.1098/rsta.2011.0081 21893527

[B32] Quandt F. Hummel F. C. (2014). The influence of functional electrical stimulation on hand motor recovery in stroke patients: a review. Exp. Transl. Stroke Med. 6:9. 10.1186/2040-7378-6-9 25276333 PMC4178310

[B33] Reynolds C. Osuagwu B. A. Vuckovic A. (2015). Influence of motor imagination on cortical activation during functional electrical stimulation. Clin. Neurophysiol. 126, 1360–1369. 10.1016/j.clinph.2014.10.007 25454278 PMC4493293

[B34] Rupp R. Kreilinger A. Rohm M. Kaiser V. Muller-Putz G. R. (2012). “Development of a non-invasive, multifunctional grasp neuroprosthesis and its evaluation in an individual with a high spinal cord injury,” in Proceedings of the 2012 Annual International Conference of the IEEE Engineering in Medicine and Biology Society (New York, NY: IEEE), 1835–1838. 10.1109/EMBC.2012.6346308 23366269

[B35] Rushton D. N. (2003). Functional electrical stimulation and rehabilitation—an hypothesis. Med. Eng. Phys. 25, 75–78. 10.1016/s1350-4533(02)00040-1 12485788

[B36] Sasaki R. Kotan S. Nakagawa M. Miyaguchi S. Kojima S. Saito K.. (2017). Presence and absence of muscle contraction elicited by peripheral nerve electrical stimulation differentially modulate primary motor cortex excitability. Front. Hum. Neurosci. 11:146. 10.3389/fnhum.2017.00146 28392766 PMC5364169

[B37] Sayenko D. G. Nguyen R. Popovic M. R. Masani K. (2014). Reducing muscle fatigue during transcutaneous neuromuscular electrical stimulation by spatially and sequentially distributing electrical stimulation sources. Eur. J. Appl. Physiol. 114, 793–804. 10.1007/s00421-013-2807-4 24390690 PMC3950614

[B38] Smith G. V. Alon G. Roys S. R. Gullapalli R. P. (2003). Functional MRI determination of a dose-response relationship to lower extremity neuromuscular electrical stimulation in healthy subjects. Exp. Brain Res. 150, 33–39. 10.1007/s00221-003-1405-9 12698214

[B39] Thorsen R. Binda L. Chiaramonte S. Dalla Costa D. Redaelli T. Occhi E.. (2014). Correlation among lesion level, muscle strength and hand function in cervical spinal cord injury. Eur. J. Phys. Rehabil. Med. 50, 31–38. 23820875

[B40] Wegrzyk J. Ranjeva J.-P. Fouré A. Kavounoudias A. Vilmen C. Mattei J. P.. (2017). Specific brain activation patterns associated with two neuromuscular electrical stimulation protocols. Sci. Rep. 7:2742. 10.1038/s41598-017-03188-9 28577338 PMC5457446

